# Cold Atmospheric Plasma in the Treatment of Autoimmune Diseases: Mechanisms, Applications, and Prospects

**DOI:** 10.1002/iid3.70245

**Published:** 2025-08-13

**Authors:** Zhenyu Li, Minglong Cai, Zhu Chen

**Affiliations:** ^1^ Department of Rheumatology and Immunology, The First Affiliated Hospital of USTC, Division of Life Sciences and Medicine University of Science and Technology of China Hefei China

**Keywords:** autoimmune disease, cold atmospheric plasma, inflammation, treatment

## Abstract

**Background:**

Autoimmune diseases, including rheumatoid arthritis (RA), are characterized by an aberrant immune responses that leads to chronic inflammation and tissue damage. Traditional treatments, such as immunosuppressive drugs, only provide symptomatic relief and often cause significant side effects. Cold atmospheric plasma (CAP), a form of nonthermal plasma, has emerged as a potential therapeutic tool, offering antimicrobial, anti‐inflammatory, and immune‐modulatory effects.

**Objective:**

This review aims to explore the mechanisms of CAP, its application in autoimmune diseases, and its potential to improve existing treatments.

**Methods:**

The review synthesizes recent studies investigating the biological effects of CAP, particularly its interaction with immune cells. Key mechanisms discussed include the generation of reactive oxygen and nitrogen species (ROS/RNS), which modulate immune responses, promote wound healing, and target pathogenic cells. The therapeutic potential of CAP in treating autoimmune diseases, such as RA, atopic dermatitis, allergic contact dermatitis, psoriasis, and vitiligo is examined through current research findings.

**Results:**

Studies have demonstrated that CAP can modulate fibroblast‐like synoviocytes in RA, reducing their viability and inducing apoptosis. In skin diseases like atopic dermatitis, CAP has been shown to alleviate symptoms and reduce microbial load by altering the skin microbiome. In psoriasis, CAP suppresses Th17 cell differentiation and reduces keratinocyte hyperproliferation. Additionally, CAP enhances wound healing by promoting macrophage M2 polarization and collagen remodeling. Despite promising results, concerns remain about the long‐term safety of CAP, particularly regarding the accumulation of ROS/RNS.

**Conclusion:**

CAP offers a novel approach for treating autoimmune diseases by modulating immune responses, enhancing drug efficacy, and promoting tissue repair. Its ability to selectively target pathogenic cells and its antimicrobial properties make it a promising therapeutic tool in autoimmune diseases.

## Graphical abstract

This review explores cold atmospheric plasma (CAP) as an emerging therapeutic strategy for autoimmune diseases. CAP has shown potential in modulating immune responses, targeting pathogenic cells, and enhancing wound healing, offering promise for autoimmune disease management with reduced side effects. The article discusses CAP's mechanisms, applications, and the need for further research to assess its long‐term safety and efficacy.

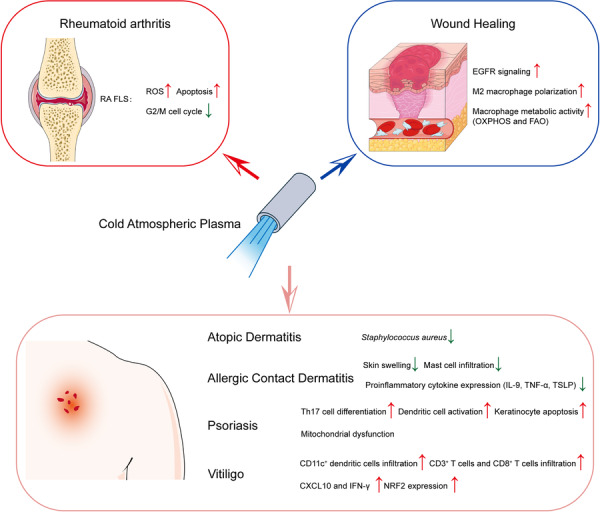

## 1 Introduction

Autoimmune diseases are characterized by the immune system's aberrant response mistakenly attacking one's own body, leading to chronic inflammation, tissue damage, and a broad spectrum of clinical manifestations, which include Rheumatoid arthritis (RA), Graves' disease, Hashimoto's thyroiditis, and Sjogren's syndrome, which each affect about 1% of the world's population [1]. Current treatments often involve immunosuppressive drugs or biological agents, which, although practical, can lead to significant side effects, including increased susceptibility to infections and other long‐term health issues. Moreover, these treatments often provide only symptomatic relief and do not address the underlying causes of the diseases, highlighting the need for novel therapeutic approaches that are both effective and have a reduced risk of adverse effects.

Plasma, often called the fourth state of matter, has emerged as a promising tool in various biomedical applications. Plasma is generated when a gas is energized to the point where its molecules or atoms become excited and ionized, resulting in a mixture of positive ions, free electrons, and reactive neutral species. This unique composition imparts plasma with properties that can be harnessed for therapeutic purposes [2]. Among various forms, cold atmospheric plasma (CAP), also known as nonthermal or cold plasma, has gained considerable attention due to its potential applications in medicine, particularly in treating chronic wounds, infections, and even cancer [3, 4, 5, 6, 7, 8, 9, 10, 11]. This review aims to explore the prospects of plasma technology in the treatment of autoimmune diseases, focusing on its mechanisms of action, current research findings, and future potential. In this review, we first introduce the fundamentals of plasma technology (Section 2), followed by mechanistic insights into CAP's immunoregulatory effects (Section 3). Section 4 discusses CAP's current applications in various autoimmune and immunological diseases, while Section 5 outlines future directions in the application of CAP for autoimmune diseases. Section 6 discusses the challenges in the application of CAP for clinic. Finally, Section 7 provides a summary and concluding remarks.

## 2 Overview of Plasma Technology

### 2.1 Plasma Composition and Biological Activity

**Figure 1:**
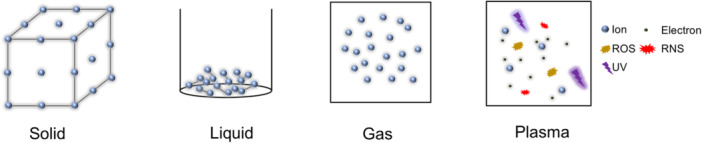
States of matter: Solid, liquid, gas, and plasma. This diagram illustrates the four fundamental states of matter: solid, liquid, gas, and plasma. Plasma is characterized by a mixture of ions, electrons, ultraviolet (UV), reactive oxygen species (ROS) and reactive nitrogen species (RNS).

Plasma is a highly energized state of matter distinct from the solid, liquid, and gas phases (Figure 1). CAP is composed of a complex mixture of charged particles, electric fields, ultraviolet radiation, and reactive oxygen and nitrogen species (ROS/RNS), including hydroxyl radicals (OH), hydrogen peroxide (H₂O₂), singlet oxygen (¹O₂), superoxide anion (O₂⁻), nitric oxide (NO), and peroxynitrite (ONOO⁻) [12, 13, 14]. The biological effects of CAP are primarily mediated by these reactive species, which induce redox signaling, modulate immune responses, and exert antimicrobial and anticancer effects [15, 16].

The physiological impact of these species is dose‐dependent: low concentrations promote immune cell activation, proliferation, and tissue repair, whereas high levels lead to oxidative damage, mitochondrial dysfunction, and apoptosis [17]. Among these, ¹O₂ and •OH are highly potent oxidative agents, inducing lipid peroxidation and catalase inactivation in bacterial and tumor cells [18, 19, 20]. Superoxide and H₂O₂ play central roles in the elimination of microbial and cancer cells through oxidative burst mechanisms [21, 22, 23]. NO, a major RNS in CAP, exerts broad immunoregulatory and wound healing functions by promoting vasodilation, regulating fibroblast and keratinocyte activity, and inhibiting bacterial DNA synthesis [12, 24, 25, 26, 27, 28, 29].

### 2.2 Delivery Challenges

Despite its promising biological effects, CAP's direct penetration into tissues is limited—studies show an in vivo depth of approximately 400 µm [30]. This shallow penetration poses challenges for deep tissue applications, such as joints or internal mucosal surfaces. Electric field exposure has been shown to stimulate fibroblast migration, keratinocyte differentiation, angiogenesis, and accelerated wound healing [31, 32]. Electroporation also facilitates molecular translocation across cell membranes and improves delivery of reactive species and therapeutic agents [33].

The generation and composition of CAP are highly dependent on gas mixture, humidity, and device parameters, which influence the yield of specific radicals such as OH, NO, and H₂O₂ [34, 35, 36]. Thus, careful modulation of CAP parameters is essential for balancing efficacy and safety in medical applications. Additionally, the presence of biological molecules (e.g., proteins, lipids, ions) in vivo alters CAP chemistry and requires complex modeling beyond simple water or saline systems.

Although CAP is a relatively recent entrant to the biomedical field, it has generated increasing global interest. Encouraging in vivo results have led to the CE certification of several CAP devices for medical use in Europe, highlighting their clinical potential. Importantly, current evidence indicates that plasma therapies are generally well‐tolerated with minimal adverse effects. Nonetheless, in the context of autoimmune diseases, potential risks—particularly related to immune overactivation or tissue damage—require careful evaluation. These considerations underscore the promise of CAP as a safe and effective therapeutic strategy, while also emphasizing the need for further mechanistic and clinical studies to fully establish its safety profile in immune‐related conditions.

## 3 Mechanistic Insights Into CAP in Immune Regulation

CAP can influence immune responses through a variety of biological mechanisms, primarily mediated by ROS and RNS, electromagnetic fields, and UV radiation. These bioactive agents modulate inflammatory signaling, immune cell behavior, and cell survival in both innate and adaptive immune contexts. CAP‐generated ROS and RNS can activate redox‐sensitive transcription factors such as NF‐κB and Nrf2, thereby regulating inflammatory and antioxidant gene expression. For instance, CAP treatment induces intracellular and mitochondrial ROS in keratinocytes, leading to upregulation of interleukin (IL)‐6, IL‐8, tumor necrosis factor (TNF)‐α and vascular endothelial growth factor (VEGF), while also reducing IL‐12 levels‐indicating both proapoptotic and immunomodulatory functions [37].

T helper 17 (Th17) cell differentiation and dendritic cell activation‐key drivers in autoimmune skin diseases like psoriasis‐are notably suppressed after CAP or plasma‐activated liquid treatment [38]. CAP has also been shown to enhance PD‐L1 expression in HaCaT keratinocytes cells, providing a potential mechanism to dampen T cell overactivation via checkpoint pathways [38].

CAP also exerts direct proapoptotic effects on hyperproliferative epithelial cells. In psoriasis models, CAP or plasma‐activated media (PAM) induces ROS‐mediated mitochondrial membrane potential loss and apoptosis in keratinocytes, contributing to the resolution of psoriatic plaques [39, 40]. Beyond epithelial tissues, CAP has demonstrated therapeutic potential in RA by directly targeting fibroblast‐like synoviocytes (RA‐FLS), which play a pivotal role in synovial inflammation and joint destruction. CAP treatment has been shown to induce mitochondrial ROS accumulation in RA‐FLS, leading to apoptosis and suppression of inflammatory infiltration in RA joints [41]. Additionally, CAP can induce G2/M phase cell cycle arrest in RA‐FLS and reduce their migration and invasion abilities—features that mirror tumor‐like behavior in the rheumatoid synovium. These effects are closely associated with intracellular oxidative stress and indicate CAP's potential as a targeted immuno‐modulatory and antiproliferative therapy in RA [42].

In the context of macrophage biology, CAP can reprogram macrophage polarization from a pro‐inflammatory M1 phenotype to a tissue‐repairing M2 phenotype in vivo. This is achieved through modulation of metabolic pathways such as oxidative phosphorylation (OXPHOS) and fatty acid oxidation (FAO), as shown in CAP‐treated gelatin scaffolds promoting wound healing [43].

Overall, the immunoregulatory actions of CAP are multifaceted, dose‐ and context‐dependent, and offer promising therapeutic potential in autoimmune and inflammatory disorders by modulating cytokine profiles, redox balance, immune checkpoint molecules, and cellular phenotypes. These properties not only make CAP intriguing but also hold the potential to significantly impact the treatment of conditions that involve complex immune responses, such as autoimmune diseases, keeping the audience engaged in the discussion.

## 4 Current Research of CAP in Immunological Diseases

CAP has emerged as a novel therapeutic tool in treating immunological diseases due to its ability to modulate immune responses and promote healing. This section reviews current research highlighting CAP's potential in treating autoimmune diseases and chronic inflammatory conditions.

### 4.1 Rheumatoid Arthritis

Rheumatoid arthritis is a chronic autoimmune disease characterized by persistent inflammation of the synovial joints, leading to joint damage, pain, and reduced mobility [44, 45]. Traditional treatments, such as nonsteroidal anti‐inflammatory drugs (NSAIDs) and disease‐modifying antirheumatic drugs (DMARDs), often come with significant side effects and limited efficacy in preventing disease progression [46].

Recent studies have explored the use of CAP as a novel therapeutic strategy for RA. CAP has shown potential in modulating immune responses and directly targeting RA‐FLS, which are critical in the pathogenesis of RA. Chengbiao Ding et al. reported that CAP treatment significantly reduced RA‐FLS viability, increased production of ROS, and induced apoptosis through the mitochondrial pathway. These effects were associated with a decrease in synovial hyperplasia and inflammatory infiltration, suggesting that CAP might help alleviate both the symptoms and underlying causes of RA. Understanding these specific mechanisms of action is crucial for further research and development in the treatment of RA [41].

Furthermore, recent research by Le‐Ying Ni and colleagues has provided additional insights into how CAP inhibits the tumor‐like biological behavior of RA‐FLS. Their study demonstrated that CAP effectively induces G2/M cell cycle arrest in RA‐FLS, increasing intracellular ROS levels and promoting apoptosis. CAP treatment significantly reduced the migration and invasion capabilities of RA‐FLS, which are known for their aggressive, tumor‐like characteristics, such as fostering angiogenesis and invading surrounding tissues [42]. These findings suggest that CAP could be a promising targeted therapeutic strategy for inhibiting the progression of RA by directly impacting the pathological behavior of RA‐FLS.

### 4.2 Atopic Dermatitis

Atopic dermatitis (AD) is a multifaceted, chronic relapsing inflammatory skin disease [47, 48]. In the study of Mi Young Lee and colleagues, CAP treatment of three sessions weekly, 10 min per session alleviated the clinical severity of dermatitis symptoms and reduced post‐inflammatory hyperpigmentation without causing severe side effects [49]. Besides, Young Jae Kim and colleagues included 22 patients with mild to moderate atopic dermatitis. They found that the modified atopic dermatitis antecubital severity and eczema area and severity index scores decreased significantly in the CAP‐treated group. Additionally, patients experienced improved scores on pruritic visual analog scales, indicating reduced itchiness [50]. Moreover, microbiome analysis revealed a significant reduction in the proportion of *Staphylococcus aureus*, a common skin pathogen in AD, in the CAP‐treated group [50]. The antibacterial properties of CAP and its ability to modulate immune responses make it particularly useful in managing skin conditions where infection and inflammation are prevalent.

### 4.3 Allergic Contact Dermatitis

Allergic contact dermatitis (ACD) is a common inflammatory skin disorder triggered by exposure to exogenous allergens or irritants. Clinically, it is characterized by erythema, edema, pruritus, and rash [51, 52]. CAP has demonstrated notable anti‐inflammatory and immunoregulatory properties in ACD. Plasma‐activated AVC hydrogels, capable of storing and releasing ROS and RNS, have been shown to significantly reduce symptoms such as skin swelling, mast cell infiltration, and pro‐inflammatory cytokine expression (IL‐9, TNF‐α, TSLP) in murine models without inducing systemic toxicity [53].

### 4.4 Psoriasis

Psoriasis is a chronic immune‐mediated skin disease characterized by epidermal hyperproliferation and immune dysfunction [54, 55]. CAP has shown great promise in treating psoriasis through multiple mechanisms. In a study by Lu Gan et al., PAM induced intracellular ROS production and triggered apoptosis in inflamed keratinocytes, while atmospheric pressure plasma jet application in imiquimod (IMQ)‐induced psoriasis‐like mice alleviated skin lesions [39]. Yun Sang Lee et al. demonstrated that CAP suppressed Th17 cell differentiation and dendritic cell activation in vitro, while also enhancing PD‐L1 expression in keratinocytes. In vivo, CAP reduced epidermal hyperplasia and inflammation in IMQ‐induced murine models [38]. Tong Wu et al. developed plasma‐activated ice microneedle patches that enabled efficient transdermal RONS delivery. These patches induced mitochondrial dysfunction and keratinocyte apoptosis, effectively reducing inflammation in IMQ‐induced psoriasis‐like dermatitis models [40].

### 4.5 Vitiligo

Vitiligo is the most common acquired depigmenting disorder of the skin. It is characterized by the selective destruction of melanocytes, leading to progressive loss of pigmentation in affected areas [56, 57]. In vitiligo, CAP treatments reduced the infiltration of CD11c^+^ dendritic cells, CD3^+^ T cells and CD8^+^ T cells and levels of CXCL10 and IFN‐γ while enhancing NRF2 expression and promoting repigmentation. Clinical trials have shown partial to complete responses in focal lesions with no significant side effects [58].

### 4.6 Wound Healing

Beyond autoimmune diseases, CAP has shown promise in promoting the healing of chronic wounds, often seen in patients with compromised immune systems. CAP enhances wound healing by stimulating collagen remodeling, reducing bacterial load, and improving local immune responses [59]. For example, its use in treating chronic venous leg ulcers has significantly reduced ulcer size and faster healing rates compared to standard wound care alone. Recently, Yanfen Zheng et al. developed a novel handheld CAP device integrated with a glucose oxidase/catalase nanogel (GCN), achieving enhanced oral ulcer healing in both in vitro and in vivo models. The CAP + GCN combination not only accelerated epithelial repair but also modulated immune responses via EGFR signaling activation, demonstrating superior efficacy over conventional treatments like Watermelon Frost [60].

In addition to its antimicrobial and regenerative effects, controlled CAP delivery systems have emerged as a solution to overcome the limitations of traditional CAP therapy, such as the need for repeated treatment and the risk of oxidative damage. Nian Zhang et al. developed a gelatin‐based scaffold (CAP‐GS) that enables sustained and localized release of ROS and RNS, effectively accelerating wound healing without repeated CAP applications. Notably, CAP‐GS promoted M2 macrophage polarization, reduced local inflammation, and enhanced macrophage metabolic activity including OXPHOS and FAO—key features of the tissue‐repairing phenotype. These macrophages also secreted more growth factors, contributing to cell proliferation and tissue regeneration [43].

**Figure 2:**
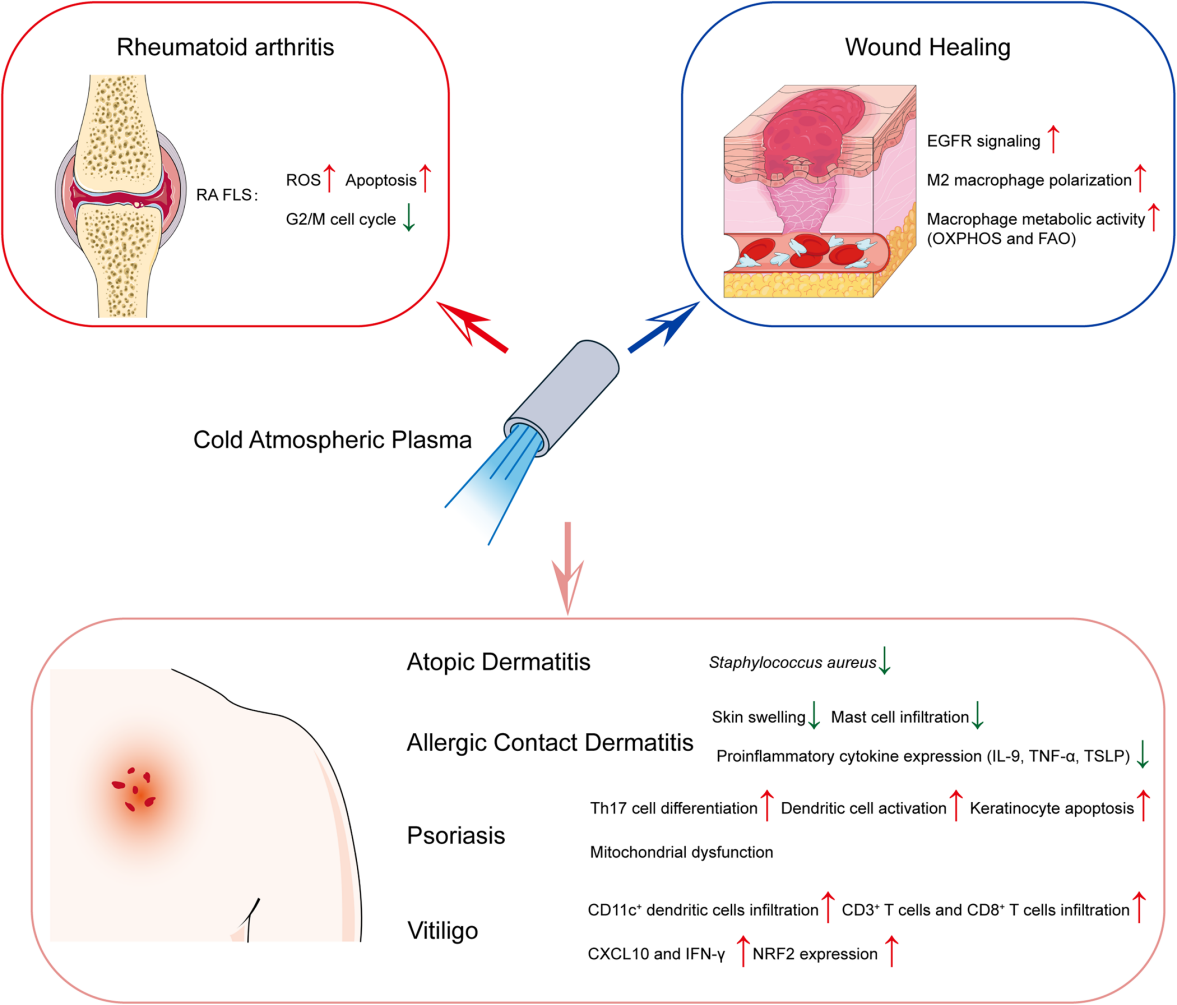
The immunoregulatory and therapeutic mechanisms of cold atmospheric plasma (CAP) in immune‐related diseases. CAP generates a variety of ions, electrons, ultraviolet (UV), reactive oxygen species (ROS) and reactive nitrogen species (RNS), which together modulate the local tissue environment and cellular behavior. In rheumatoid arthritis (RA), CAP induces apoptosis in RA fibroblast‐like synoviocytes (RA‐FLS), promotes G2/M cell cycle arrest, and inhibits migration and invasion. In atopic dermatitis (AD) and allergic contact dermatitis (ACD), CAP reduces skin inflammation by suppressing mast cell infiltration, lowering cytokine levels (e.g., IL‐9, TSLP, TNF‐α), and rebalancing skin microbiota. In psoriasis, CAP inhibits Th17 cell differentiation, reduces keratinocyte hyperproliferation, and alleviates skin lesions. In vitiligo, CAP downregulates IFN‐γ and CXCL10 expression, limits CD3⁺ and CD8⁺ T cell infiltration, and enhances NRF2‐mediated antioxidant defense, promoting melanocyte survival and repigmentation. In chronic wounds, CAP enhances macrophage M2 polarization, improves macrophage metabolic activity (OXPHOS, FAO), promotes collagen remodeling, and accelerates tissue repair. This figure was created by the authors using adapted elements from Servier Medical Art (https://smart.servier.com/), licensed under CC BY 4.0 (https://creativecommons.org/licenses/by/4.0/).

Taking Together, the application of CAP in treating immunological diseases, particularly autoimmune disorders like rheumatoid arthritis, presents a novel and promising approach. Its ability to modulate immune responses, selectively target pathological cells and promote healing without significant side effects positions. As summarized in Table 1 and Figure 2, CAP has demonstrated diverse therapeutic applications across a range of autoimmune and immune‐related disorders. In rheumatoid arthritis, CAP effectively induces apoptosis of RA‐FLS both in vitro and in vivo, thereby mitigating synovial hyperplasia and joint inflammation [41, 42]. In AD and psoriasis, CAP significantly alleviates symptoms such as pruritus, erythema, and epidermal hyperproliferation. These effects are mediated by suppression of Th17 cell differentiation, reduction of keratinocyte proliferation, and modulation of the skin microbiome [37, 38, 39, 40, 49, 50].

**Table 1:** CAP applications in autoimmune and immune‐related disorders.

Disease/Condition	CAP form used	Mechanism of action	Therapeutic outcome	Model type	Reference
Rheumatoid arthritis (RA)	Direct CAP	Induce RA‐FLS ROS ↑, mitochondria dysfunction, G2/M cell cycle arrest, apoptosis↑	Alleviate the symptoms of RA	In vitro, In vivo	[41, 42]
Atopic dermatitis	Direct CAP	Reduction in the proportion of *Staphylococcus aureus*	Reduces severity, pruritus, and bacterial load	Clinical	[49, 50]
Allergic contact dermatitis	Plasma‐activated AVC hydrogel	Mast cell infiltration ↓; pro‐inflammatory cytokine expression (IL‐9, TNF‐α, TSLP)↓	Reduce skin swelling	In vivo	[53]
Psoriasis	Direct CAP, plasma‐activated medium, plasma‐activated Ice microneedle patches	Induce keratinocyte mitochondrial dysfunction, apoptosis ↑, PD‐L1 ↑; Th17 cell differentiation↓	Alleviate skin lesions	In vitro, In vivo	[37, 38, 39, 40]
Vitiligo	Direct CAP, CAP hydrogel	Infiltration of CD11c dendritic cells ↓, CD3 T cells ↓； CD8 T cells ↓ ; CXCL10 ↓ IFN‐γ ↓ ; NRF2 ↑ ; Repigmentation↑	Repigmentation	In vivo, Clinical	[58]
Wound healing	Plasma‐activated medium, CAP with a skin bionics gelatin scaffold, Nanogel‐based pharmaceuticals	M2 macrophage polarization ↑, macrophage metabolic activity including OXPHOS and FAO ↑, EGFR signaling↑	Reduce pain and promote healing	In vivo, Clinical	[43, 59, 60]

The use of plasma‐activated materials, such as AVC hydrogels and microneedle patches, enables localized and sustained delivery of ROS/RNS, enhancing CAP's therapeutic efficacy in diseases like allergic contact dermatitis and chronic wounds [43, 53, 59]. Moreover, in vitiligo, CAP reduces immune cell infiltration and inflammatory cytokines, while upregulating NRF2, promoting melanocyte survival and skin repigmentation [58].

These disease‐specific outcomes illustrate CAP's multifaceted roles in immune regulation, tissue repair, antimicrobial defense, and selective cytotoxicity, reinforcing its potential as a novel and versatile treatment strategy for autoimmune diseases.

## 5 Future Prospects in the Application of CAP for Autoimmune Diseases

**Figure 3:**
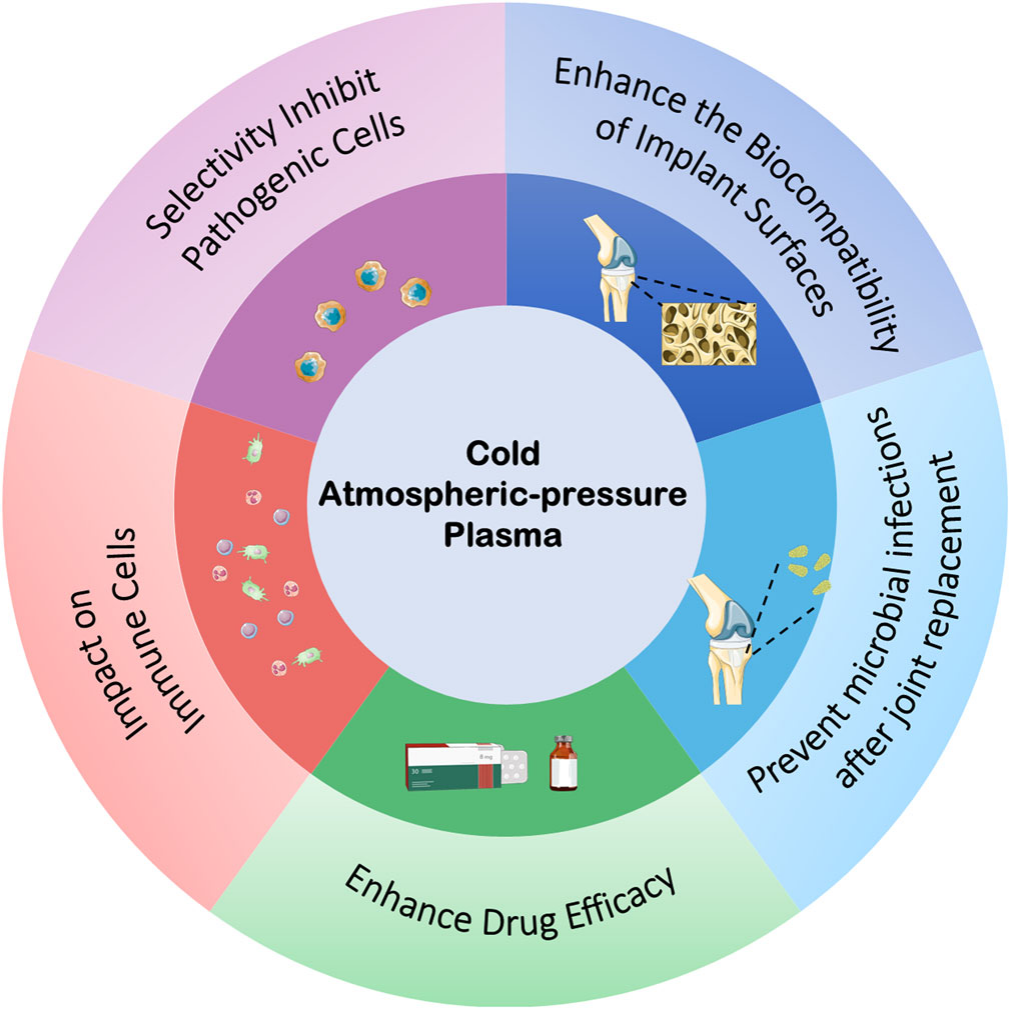
Prospects of cold atmospheric plasma (CAP) therapy for autoimmune diseases. This diagram illustrates five potential directions for applying CAP in the treatment of autoimmune diseases: (1) Enhance the biocompatibility of implants—improving tissue integration and reducing the risk of implant loosening. (2) Prevent microbial infections after joint replacement—leveraging CAP's antimicrobial properties to reduce biofilm formation. (3) Enhance drug efficacy—increasing immune cell sensitivity to conventional therapies. (4) Impact on Immune Cells—adjusting cytokine profiles and cell surface markers to reduce inflammation. (5) Selectivity Inhibit Pathogenic Cells—inducing apoptosis in hyperactive or autoreactive immune cells while sparing normal tissue. This figure was created by the authors using adapted elements from Servier Medical Art (https://smart.servier.com/), licensed under CC BY 4.0 (https://creativecommons.org/licenses/by/4.0/).

The use of CAP in treating autoimmune diseases, such as RA, holds significant promise. However, several challenges and areas of uncertainty need to be addressed to optimize its therapeutic potential (Figure 3).

### 5.1 Prevention of Complications Post‐Joint Replacement Surgery of RA

Joint replacement surgery is a standard treatment for severe RA to alleviate pain and restore function. However, these procedures are often accompanied by complications such as periprosthetic joint infections and implant loosening, which can lead to significant morbidity and necessitate revision surgeries [61, 62]. The incidence of these complications is exceptionally high in RA patients due to chronic inflammation and immune dysregulation [63, 64]. These complications increase healthcare costs and significantly affect patients' quality of life for patients [65].

CAP has emerged as a promising strategy to prevent such complications by enhancing implant surfaces' biocompatibility and antibacterial properties. CAP treatment can improve the hydrophilicity of implant surfaces, promoting better integration with surrounding tissues. For instance, the implaPrep prototype device, which utilizes CAP via DBD, demonstrated that titanium surfaces treated with CAP showed increased cell adhesion and viability compared to untreated surfaces [66]. This enhancement in biocompatibility could potentially reduce the risk of implant loosening by improving tissue integration.

Moreover, CAP has been shown to retain the nanostructure of implant materials like Ti6Al4V while promoting osteogenic differentiation of bone marrow–derived mesenchymal stem cells (BM‐MSCs), enhancing bone healing and implant stability [67]. Studies using air CAP on titanium surfaces have demonstrated improved cell proliferation and migration, as well as upregulation of genes related to cell adhesion (e.g., integrin β1, α2 and α5) [68]. These findings suggest that CAP treatment of implants could improve initial cellular attachment, cell proliferation, and overall osseointegration, which are critical for the long‐term success of joint replacements.

CAP also exhibits potent antimicrobial properties crucial in preventing periprosthetic joint infections. In vitro, studies have shown that CAP treatment effectively eradicates biofilms formed by common pathogens such as *Staphylococcus aureus* and *Escherichia coli*, which are frequently involved in prosthetic joint infections [69, 70]. Simultaneously eradicating biofilm contamination and enhancing surface properties to inhibit microbial recolonization, CAP‐treated implants could benefit infection control and biocompatibility enhancement.

Furthermore, CAP has been studied for its ability to improve bone healing around implants. Studies using nitrogen plasma show that short‐time plasma treatment can promote osteogenic differentiation of preosteoblasts, essential for bone regeneration and implant stability [71]. In the context of peri‐implantitis, a condition that severely impedes osseointegration, CAP treatment has been shown to increase the roughness and hydrophilicity of titanium surfaces, enhance bone formation, and provide effective sterilization [72].

These studies collectively highlight the potential of CAP as a preventative strategy against common complications associated with joint replacement surgery in RA patients. By improving both the biocompatibility and antimicrobial properties of implant surfaces, CAP could significantly reduce the incidence of infections and implant loosening, leading to better clinical outcomes and enhanced quality of life for patients undergoing joint replacement. Future research should focus on clinical trials to validate these findings and optimize CAP treatment protocols for orthopedic implants.

### 5.2 Enhancing Drug Efficacy

CAP has shown a potential to enhance the efficacy of conventional drug treatments, which is a promising area of research that is particularly relevant for autoimmune diseases. In cancer therapy, CAP has been found to increase drug sensitivity in drug‐resistant cancer cells by generating ROS and RNS, which can potentiate the effects of anticancer drugs [73, 74]. This synergistic effect raises the possibility that similar mechanisms could be harnessed to improve the efficacy of pharmacological treatments in autoimmune disorders.

In studies on cancer cells, plasma‐activated solutions (PAS) and PAM have been used to enhance the cytotoxic effects of drugs like cisplatin, doxorubicin, trametinib, and sorafenib. These studies demonstrated that CAP and PAM could induce both caspase‐dependent and caspase‐independent cell death in cancer cells, thereby enhancing the overall anticancer effects [75, 76]. The increased intracellular ROS levels facilitated by CAP lead to oxidative stress, making cancer cells more susceptible to drug‐induced apoptosis. Translating this approach to autoimmune diseases could involve using CAP to increase the sensitivity of hyperactive immune cells or fibroblast‐like synoviocytes to standard anti‐inflammatory and immunosuppressive drugs.

For instance, PAS has increased intracellular ROS levels in drug‐resistant cancer cells, leading to enhanced drug sensitivity and cell death [73]. This method could be explored in autoimmune diseases to enhance the effects of drugs like methotrexate, sulfasalazine, or biologics that target specific inflammatory pathways. Modifying the inflammatory microenvironment with CAP may reduce the required dosages of these drugs, thus minimizing side effects and improving patient outcomes.

Furthermore, studies have shown that combining CAP with other therapies, such as electric field treatments, may amplify the therapeutic effects by further disrupting cellular functions [74]. This combination approach could be particularly beneficial in autoimmune diseases, where the goal is often to modulate the immune response without completely suppressing it, as excessive immunosuppression can lead to increased susceptibility to infections.

The application of CAP to enhance drug efficacy in autoimmune diseases represents an innovative strategy that warrants further investigation. The same principles that have shown promise in oncology—enhancing ROS production, increasing drug uptake, and promoting targeted cell death—could be applicable in treating RA and other autoimmune conditions. Future research should focus on elucidating the exact mechanisms by which CAP enhances drug action in autoimmune settings, optimizing treatment protocols, and conducting clinical trials to validate efficacy and safety. This approach can potentially revolutionize the management of autoimmune diseases, offering patients more effective and tailored therapeutic options. However, it is important to note that most current evidence supporting this application comes from oncology, and further investigation is needed to confirm these mechanisms in autoimmune contexts.

### 5.3 Impact on Immune Cells

The interaction of CAP with immune cells is a crucial area for future research, particularly regarding its potential to modulate immune responses in autoimmune diseases. CAP has demonstrated effects on immune cell surface markers and cytokine profiles, which could influence inflammatory responses and disease progression. For example, studies have shown that CAP exposure can alter the expression of adhesion molecules such as l‐selectin, ICAM‐1, and LFA‐1α on T cells, potentially modifying their activity and homing behavior [77].

Additionally, recent research has indicated that CAP can significantly impact immune responses in conditions such as atopic dermatitis. In a clinical study, CAP treatment was found to alleviate the clinical severity of atopic dermatitis and reduce the proportion of *Staphylococcus aureus* in skin lesions, suggesting a role in modulating both immune response and microbial presence on the skin [50]. This highlights CAP's potential to reduce inflammation and alter the local immune environment to manage autoimmune skin conditions better.

Further studies have explored CAP's ability to enhance antitumor immune responses. For instance, CAP treatment in a postsurgical cancer setting was shown to induce immunogenic cell death, which in turn triggered strong T cell‐mediated immune responses against residual tumor cells [78]. This finding suggests that CAP might similarly enhance immune responses in autoimmune diseases by promoting an environment conducive to immunomodulation. The capacity of CAP to stimulate immune surveillance and adjust the balance of immune cell types could be leveraged to control autoimmune reactions, reducing chronic inflammation and tissue damage.

These studies suggest that CAP's effects on immune cells could be multifaceted, potentially offering benefits in enhancing immune defense against infections and modulating overactive immune responses characteristic of autoimmune diseases. Future research should focus on understanding the precise mechanisms through which CAP influences different immune cell subsets, such as T cells, B cells, and macrophages, to better harness its therapeutic potential in managing autoimmune diseases.

### 5.4 Selectivity Towards Pathogenic Cells

One of the most compelling aspects of CAP is its demonstrated ability to selectively target pathogenic cells, such as cancer cells while sparing normal, healthy cells. This selectivity is crucial for minimizing collateral damage during treatment, which is a significant concern in both cancer therapy and autoimmune disease management [79, 80]. In cancer research, CAP has been shown to induce cell death selectively in tumor cells through mechanisms such as the generation of ROS, which lead to oxidative stress and apoptosis in cancer cells while leaving normal cells largely unaffected [75, 81].

For example, studies have demonstrated that PAM can induce apoptosis, specifically in cancer stem cells, which are often responsible for tumor resistance and relapse. These effects have been observed in hepatocellular carcinoma cell lines, where PAM increased the efficacy of chemotherapeutic agents like cisplatin by selectively inducing cell death in cancer stem cells [75]. Similar selectivity has been noted with the use of CAP in breast cancer cells, where plasma‐activated water showed a preference for killing cancer cells over normal fibroblast cells, suggesting a potentially safer therapeutic profile [82]. A novel FlexiPlasma Microcatheter–Embolic Material platform developed by Changhong Li et al. enabled precise CAP delivery to deep tumors via noninflammatory pyroptosis, mediated through the ROS/Caspase‐8/GSDMC pathway [83]. Although designed for hepatocellular carcinoma, this selective cytotoxicity and immunostimulatory effect suggest potential for extending CAP to autoreactive immune cell targeting in autoimmune diseases.

Autoimmune diseases, such as RA, involve various pathogenic immune cells, including T cells, B cells, macrophage, and RA‐FLS, contributing to chronic inflammation and tissue damage. Whether CAP can selectively target these disease‐causing cells while sparing healthy immune cells remains unanswered. Understanding this selectivity is crucial for translating CAP into clinical applications for autoimmune diseases.

In autoimmune contexts, CAP's selectivity could theoretically help modulate pathological immune responses by targeting and eliminating overactive or autoreactive immune cells without compromising the overall immune system's integrity. For example, CAP could induce apoptosis in RA‐FLS, similar to its effects on cancer cells, thereby reducing synovial hyperplasia and inflammation in RA [79]. However, the safety and efficacy of CAP in selectively targeting these pathogenic cells need thorough investigation through preclinical and clinical studies.

Given the success of CAP in selectively targeting cancer cells and the shared mechanisms of abnormal cell proliferation and survival between cancer and autoimmune diseases, there is a strong rationale for further exploring CAP's potential selectivity in autoimmune treatment. Research should focus on identifying the specific molecular pathways CAP affects in autoimmune cells and establishing whether these effects can be harnessed to inhibit disease‐driving immune responses while preserving normal immune function selectively. This approach could provide a novel therapeutic strategy to manage autoimmune diseases more effectively and safely.

## 6 Challenges in the Application of CAP for Clinic

CAP has shown significant therapeutic potential, particularly in the treatment of chronic wounds and infectious conditions. CAP's ability to promote wound healing, reduce microbial load, and alleviate pain has been well‐documented across various studies, including its application in pressure ulcers [84], diabetic foot ulcers [85], and superficial skin erosions [86]. However, its clinical use requires careful consideration of both its benefits and potential risks, especially in long‐term treatments.

CAP has demonstrated efficacy in various wound healing scenarios, with studies suggesting that both once‐weekly and twice a week can result in significant wound healing and pain reduction [87]. This raises the question of optimizing treatment frequency to balance therapeutic outcomes with patient convenience and healthcare resource management.

Equally important is the consideration of CAP's safety profile, particularly concerning the generation of ROS and RNS. These reactive species are central to CAP's therapeutic mechanisms; however, excessive or sustained exposure may induce oxidative stress and cause unintended damage to healthy tissues. Although CAP has been proven safe in short‐term applications in current clinical trials [84, 87], its long‐term safety, particularly concerning the accumulation of ROS and RNS, warrants further investigation.

In conclusion, while CAP offers considerable promise in wound healing and infection control, caution is necessary regarding its long‐term application due to the potential side effects of ROS and RNS on healthy tissues. Although early studies support the clinical reliability of CAP [84, 85, 86, 87], further research is required to fully understand its impact on the immune system, particularly in autoimmune diseases. Comprehensive preclinical and clinical studies are essential to assess CAP's long‐term safety and explore its therapeutic potential in broader medical contexts, including autoimmune disorders.

## 7 Conclusion

CAP has emerged as a promising therapeutic tool with potential applications in treating autoimmune diseases. Its ability to enhance implants' biocompatibility and antimicrobial properties suggests that CAP can significantly reduce complications like infections and implant loosening following joint replacement surgeries. Moreover, CAP's capacity to enhance the efficacy of conventional drugs by modifying the inflammatory microenvironment opens new avenues for improving existing treatment protocols for autoimmune conditions. CAP could help minimize adverse side effects associated with long‐term immunosuppressive therapies by lowering drug doses while maintaining therapeutic effectiveness.

Another intriguing aspect of CAP is its demonstrated selectivity towards pathogenic cells, which has been well‐documented in cancer research. However, the extension of this selectivity to autoimmune diseases remains an area of active exploration. The potential for CAP to selectively target autoreactive immune cells and FLS offers a novel approach to modulating pathological immune responses without compromising overall immune function. While preliminary studies have shown promising results, further research is needed to fully understand CAP's mechanisms of action, optimize treatment protocols, and ensure its safety and efficacy in clinical settings. To advance this promising field, future studies should aim to: (1) Identify specific molecular targets of CAP in autoimmune cells; (2) Validate long‐term safety profiles in autoimmune models; (3) Conduct clinical trials assessing efficacy in diverse autoimmune diseases. As research progresses, CAP may become an integral part of the therapeutic arsenal against autoimmune diseases, providing patients with safer, more effective treatment options and improving their quality of life.

## Author Contributions

All authors contributed to the design of the article. **Zhenyu Li:** conceptualization, data curation, visualization, writing original draft. **Minglong Cai:** conceptualization, writing – review and editing. **Zhu Chen:** conceptualization, writing – review and editing, supervision. All authors read and approved the final manuscript.

## Conflicts of Interest

The authors declare no conflicts of interest.

## Data Availability

The authors have nothing to report.

## References

[1] J. H. Cho and M. Feldman , “Heterogeneity of Autoimmune Diseases: Pathophysiologic Insights From Genetics and Implications for New Therapies,” Nature Medicine 21, no. 7 (2015): 730–738. 10.1038/nm.3897 PMC5716342 26121193

[2] Y. E. Shalom Eliezer , The Fourth State of Matter: An Introduction to Plasma Science, 2nd Edition. 2001.

[3] Y. Zhao , Y. Liu , Z. Liu , et al., “A 3D‐printed Fence‐Surface Plasma Source for Skin Treatment and Its Potential for Personalized Medical Application,” Journal of Physics D: Applied Physics 57, no. 12 (2024): 125207.

[4] X. Sheng , J. Wang , L. Zhao , et al., “Inactivation Mechanism of Cold Plasma Combined With 222 nm Ultraviolet for Spike Protein and Its Application in Disinfecting of SARS‐CoV‐2,” Journal of Hazardous Materials 465 (2024): 133458. 38215522 10.1016/j.jhazmat.2024.133458

[5] Y. Zhang , Z. Chen , C. Shao , and Q. Huang , “Study of the Effect of Shock Wave From a Portable Pulsed Cold Air Plasma Jet Device on Inactivation of Trichophyton Rubrum in Nails,” Applied Physics Letters 124, no. 24 (2024): 243701.

[6] S. Xu , X. Jing , J. Zhang , et al., “Anticancer Effects of DBD Plasma‐Activated Saline Within Different Discharge Modes,” Journal of Physics D: Applied Physics 56, no. 34 (2023): 345205.

[7] M. Suzuki‐Karasaki , Y. Ochiai , S. Innami , et al., “Ozone Mediates the Anticancer Effect of Air Plasma by Triggering Oxidative Cell Death Caused by H2O2 and Iron,” European Journal of Cell Biology 102, no. 4 (2023): 151346. 37572557 10.1016/j.ejcb.2023.151346

[8] M. Pastorek , M. Suchoňová , B. Konečná , et al., “The Effect of Air Plasma Activated Liquid on Uropathogenic Bacteria,” Plasma Chemistry and Plasma Processing 42, no. 3 (2022): 561–574.

[9] S. Peng , M. Qi , H. Zhang , et al., “Discharge Characteristics of a Microsecond Pulse Power Supply Driven Air Plasma Jet and Its Anticancer Cell Effect,” Physics of Plasmas 29, no. 1 (2022): 013504.

[10] S. K. Dubey , S. Parab , A. Alexander , et al., “Cold Atmospheric Plasma Therapy in Wound Healing,” Process Biochemistry 112 (2022): 112–123.

[11] H. Liu , X. Liang , M. Teng , et al., “Cold Atmospheric Plasma: An Emerging Immunomodulatory Therapy,” Advanced Therapeutics 7, no. 5 (2024): 2300399.

[12] C. V. Suschek and C. Opländer , “The Application of Cold Atmospheric Plasma in Medicine: The Potential Role of Nitric Oxide in Plasma‐Induced Effects,” Clinical Plasma Medicine 4, no. 1 (2016): 1–8.

[13] G. Colonna , C. D. Pintassilgo , F. Pegoraro , et al., “Theoretical and Experimental Aspects of Non‐Equilibrium Plasmas in Different Regimes: Fundamentals and Selected Applications,” European Physical Journal D 75, no. 6 (2021): 183.

[14] P. R. Sreedevi , K. Suresh , and S. Yugeswaran , “Cold Atmospheric Plasma‐Induced Oxidative Stress and Ensuing Immunological Response – a Neo‐Vista in Immunotherapy,” Free Radical Research 56, no. 7–8 (2022): 498–510. 36282274 10.1080/10715762.2022.2139691

[15] M. Weiss , G. Daeschlein , A. Kramer , et al., “Virucide Properties of Cold Atmospheric Plasma for Future Clinical Applications,” Journal of Medical Virology 89, no. 6 (2017): 952–959. 27696466 10.1002/jmv.24701

[16] M. Irshad and P. S. Chaudhuri , “Oxidant‐Antioxidant System: Role and Significance in Human Body,” Indian Journal of Experimental Biology 40, no. 11 (2002): 1233–1239. 13677624

[17] D. R. Green , L. Galluzzi , and G. Kroemer , “Mitochondria and the Autophagy–Inflammation–Cell Death Axis in Organismal Aging,” Science 333, no. 6046 (2011): 1109–1112. 21868666 10.1126/science.1201940 PMC3405151

[18] Z. Machala , B. Tarabova , K. Hensel , E. Spetlikova , L. Sikurova , and P. Lukes , “Formation of ROS and RNS in Water Electro‐Sprayed Through Transient Spark Discharge in Air and Their Bactericidal Effects,” Plasma Processes and Polymers 10, no. 7 (2013): 649–659.

[19] H. Wu , P. Sun , H. Feng , et al., “Reactive Oxygen Species in a Non‐Thermal Plasma Microjet and Water System: Generation, Conversion, and Contributions to Bacteria Inactivation—An Analysis by Electron Spin Resonance Spectroscopy,” Plasma Processes and Polymers 9, no. 4 (2012): 417–424.

[20] G. Bauer , “Cold Atmospheric Plasma and Plasma‐Activated Medium: Antitumor Cell Effects With Inherent Synergistic Potential,” Plasma Medicine 9, no. 1 (2019): 57–88.

[21] M. Hayyan , M. A. Hashim , and I. M. AlNashef , “Superoxide Ion: Generation and Chemical Implications,” Chemical Reviews 116, no. 5 (2016): 3029–3085. 26875845 10.1021/acs.chemrev.5b00407

[22] P. Huang , L. Feng , E. A. Oldham , M. J. Keating , and W. Plunkett , “Superoxide Dismutase as a Target for the Selective Killing of Cancer Cells,” Nature 407, no. 6802 (2000): 390–395. 11014196 10.1038/35030140

[23] V. S. Khodade , M. Sharath Chandra , A. Banerjee , et al., “Bioreductively Activated Reactive Oxygen Species (ROS) Generators as MRSA Inhibitors,” ACS Medicinal Chemistry Letters 5, no. 7 (2014): 777–781. 25050164 10.1021/ml5001118 PMC4094247

[24] M. B. Witte , T. Kiyama , and A. Barbul , “Nitric Oxide Enhances Experimental Wound Healing in Diabetes,” British Journal of Surgery 89, no. 12 (2002): 1594–1601. 12445072 10.1046/j.1365-2168.2002.02263.x

[25] V. Krischel , D. Bruch‐Gerharz , C. Suschek , K. D. Kröncke , T. Ruzicka , and V. Kolb‐Bachofen , “Biphasic Effect of Exogenous Nitric Oxide on Proliferation and Differentiation in Skin Derived Keratinocytes but Not Fibroblasts,” Journal of Investigative Dermatology 111, no. 2 (1998): 286–291. 9699731 10.1046/j.1523-1747.1998.00268.x

[26] A. Pinder , E. Pittaway , K. Morris , and P. James , “Nitrite Directly Vasodilates Hypoxic Vasculature via Nitric Oxide‐Dependent and ‐Independent Pathways,” British Journal of Pharmacology 157, no. 8 (2009): 1523–1530. 19594749 10.1111/j.1476-5381.2009.00340.x PMC2765305

[27] E. Džoljić , ‐ Fau , I. Grbatinić , et al., Why Is Nitric Oxide Important for Our Brain? (1971‐3274 (Electronic)). 10.11138/FNeur/2015.30.3.159 PMC4610750 26910176

[28] T. Kisch , A. Helmke , S. Schleusser , et al., “Improvement of Cutaneous Microcirculation by Cold Atmospheric Plasma (CAP): Results of a Controlled, Prospective Cohort Study,” Microvascular Research 104 (2016): 55–62. 26655582 10.1016/j.mvr.2015.12.002

[29] J. M. Schapiro , S. J. Libby , and F. C. Fang , “Inhibition of Bacterial DNA Replication by Zinc Mobilization During Nitrosative Stress,” Proceedings of the National Academy of Sciences 100, no. 14 (2003): 8496–8501. 10.1073/pnas.1033133100 PMC166257 12829799

[30] A. Privat‐Maldonado , A. Schmidt , A. Lin , et al., “ROS From Physical Plasmas: Redox Chemistry for Biomedical Therapy,” Oxidative Medicine and Cellular Longevity 2019, no. 1–29 (2019): 9062098. 31687089 10.1155/2019/9062098 PMC6800937

[31] S. Snyder , C. DeJulius , and R. K. Willits , “Electrical Stimulation Increases Random Migration of Human Dermal Fibroblasts,” Annals of Biomedical Engineering 45, no. 9 (2017): 2049–2060. 28488217 10.1007/s10439-017-1849-x

[32] J. Dubé , O. Rochette‐Drouin , P. Lévesque , et al., “Human Keratinocytes Respond to Direct Current Stimulation by Increasing Intracellular Calcium: Preferential Response of Poorly Differentiated Cells,” Journal of Cellular Physiology 227, no. 6 (2012): 2660–2667. 21882192 10.1002/jcp.23008

[33] G. Busco , E. Robert , N. Chettouh‐Hammas , J. M. Pouvesle , and C. Grillon , “The Emerging Potential of Cold Atmospheric Plasma in Skin Biology,” Free Radical Biology and Medicine 161 (2020): 290–304. 33039651 10.1016/j.freeradbiomed.2020.10.004

[34] A. V. Pipa , S. Reuter , R. Foest , and K. D. Weltmann , “Controlling the No Production of an Atmospheric Pressure Plasma Jet,” Journal of Physics D: Applied Physics 45, no. 8 (2012): 085201.

[35] J. Winter , K. Wende , K. Masur , et al., “Feed Gas Humidity: A Vital Parameter Affecting a Cold Atmospheric‐Pressure Plasma Jet and Plasma‐Treated Human Skin Cells,” Journal of Physics D: Applied Physics 46, no. 29 (2013): 295401.

[36] J. Julák , A. Hujacová , V. Scholtz , J. Khun , and K. Holada , “Contribution to the Chemistry of Plasma‐Activated Water,” Plasma Physics Reports 44, no. 1 (2018): 125–136.

[37] S. Y. Zhong , Y. Y. Dong , D. X. Liu , et al., “Surface Air Plasma‐Induced Cell Death and Cytokine Release of Human Keratinocytes in the Context of Psoriasis,” British Journal of Dermatology 174, no. 3 (2016): 542–552. 26498849 10.1111/bjd.14236

[38] Y. S. Lee , M. H. Lee , H. J. Kim , H. R. Won , and C. H. Kim , “Non‐Thermal Atmospheric Plasma Ameliorates Imiquimod‐Induced Psoriasis‐Like Skin Inflammation in Mice Through Inhibition of Immune Responses and Up‐Regulation of PD‐L1 Expression,” Scientific Reports 7, no. 1 (2017): 15564. 29138509 10.1038/s41598-017-15725-7 PMC5686068

[39] L. Gan , J. Duan , S. Zhang , et al., “Cold Atmospheric Plasma Ameliorates Imiquimod‐Induced Psoriasiform Dermatitis in Mice by Mediating Antiproliferative Effects,” Free Radical Research 53, no. 3 (2019): 269–280. 30663913 10.1080/10715762.2018.1564920

[40] T. Wu , J. Zhang , X. Jing , et al., “Multiple RONS‐Loaded Plasma‐Activated Ice Microneedle Patches for Transdermal Treatment of Psoriasis,” ACS Applied Materials & Interfaces 16, no. 35 (2024): 46123–46132. 39180585 10.1021/acsami.4c10067

[41] C. Ding , L. Ni , Q. Liu , et al., “Cold Air Plasma Improving Rheumatoid Arthritis via Mitochondrial Apoptosis Pathway,” Bioengineering & Translational Medicine 8, no. 1 (2023): e10366. 36684093 10.1002/btm2.10366 PMC9842019

[42] L. Y. Ni , C. B. Ding , J. M. Deng , Z. W. Wu , and Y. Zhou , “Cold Air Plasma Inhibiting Tumor‐Like Biological Behavior of Rheumatoid Arthritis Fibroblast‐Like Synovial Cells via G2/M Cell Cycle Arrest,” Open Access Rheumatology: Research and Reviews 16 (2024): 75–85. 38756916 10.2147/OARRR.S438536 PMC11096841

[43] N. Zhang , G. Yang , Y. Wu , et al., “Controlled Release of Cold Atmospheric Plasma by Gelatin Scaffold Enhances Wound Healing via Macrophage Modulation,” ACS Applied Materials & Interfaces 17, no. 10 (2025): 15050–15066. 40013441 10.1021/acsami.4c21635

[44] J. S. Smolen , D. Aletaha , and I. B. McInnes , “Rheumatoid Arthritis,” Lancet 388, no. 10055 (2016): 2023–2038. 27156434 10.1016/S0140-6736(16)30173-8

[45] I. B. McInnes and G. Schett , “The Pathogenesis of Rheumatoid Arthritis,” New England Journal of Medicine 365, no. 23 (2011): 2205–2219. 22150039 10.1056/NEJMra1004965

[46] A. F. Radu and S. G. Bungau , “Management of Rheumatoid Arthritis: An Overview,” Cells 10, no. 11 (2021): 2857. 34831081 10.3390/cells10112857 PMC8616326

[47] T. Bieber , “Atopic Dermatitis,” New England Journal of Medicine 358, no. 14 (2008): 1483–1494. 18385500 10.1056/NEJMra074081

[48] S. Ständer , “Atopic Dermatitis,” New England Journal of Medicine 384, no. 12 (2021): 1136–1143. 33761208 10.1056/NEJMra2023911

[49] M. Y. Lee , C. H. Won , and Y. J. Kim , “Improvement of Post‐Inflammatory Hyperpigmentation, Subsequent to Cold Atmospheric Plasma Treatment, in a Patient With Atopic Dermatitis,” Medical Lasers 9, no. 2 (2020): 187–189.

[50] Y. J. Kim , D. J. Lim , M. Y. Lee , W. J. Lee , S. E. Chang , and C. H. Won , “Prospective, Comparative Clinical Pilot Study of Cold Atmospheric Plasma Device in the Treatment of Atopic Dermatitis,” Scientific Reports 11, no. 1 (2021): 14461. 34262113 10.1038/s41598-021-93941-y PMC8280139

[51] P. L. Scheinman , M. Vocanson , J. P. Thyssen , et al., “Contact Dermatitis,” Nature Reviews Disease Primers 7, no. 1 (2021): 38. 10.1038/s41572-021-00271-4 34045488

[52] R. S. Rashid and T. N. Shim , “Contact Dermatitis,” BMJ 353 (2016): i3299. 27364956 10.1136/bmj.i3299

[53] Z. Wu , X. Jing , S. Xu , et al., “Plasma‐Activated AVC Hydrogel for Reactive Oxygen and Nitrogen Species Delivery to Treat Allergic Contact Dermatitis,” ACS Applied Materials & Interfaces 16, no. 43 (2024): 58379–58391. 39425637 10.1021/acsami.4c14006

[54] A. M. Bowcock and J. G. Krueger , “Getting under the Skin: The Immunogenetics of Psoriasis,” Nature Reviews Immunology 5, no. 9 (2005): 699–711. 10.1038/nri1689 16138103

[55] M. A. Lowes , A. M. Bowcock , and J. G. Krueger , “Pathogenesis and Therapy of Psoriasis,” Nature 445, no. 7130 (2007): 866–873. 17314973 10.1038/nature05663

[56] M. L. Frisoli , K. Essien , and J. E. Harris , “Vitiligo: Mechanisms of Pathogenesis and Treatment,” Annual Review of Immunology 38 (2020): 621–648. 10.1146/annurev-immunol-100919-023531 32017656

[57] K. Ezzedine , V. Eleftheriadou , M. Whitton , and N. van Geel , “Vitiligo,” Lancet 386, no. 9988 (2015): 74–84. 25596811 10.1016/S0140-6736(14)60763-7

[58] S. Zhai , M. Xu , Q. Li , et al., “Successful Treatment of Vitiligo With Cold Atmospheric Plasma‒Activated Hydrogel,” Journal of Investigative Dermatology 141, no. 11 (2021): 2710–2719.e6. 34029575 10.1016/j.jid.2021.04.019

[59] F. Brehmer , H. A. Haenssle , G. Daeschlein , et al., “Alleviation of Chronic Venous Leg Ulcers With a Hand‐Held Dielectric Barrier Discharge Plasma Generator (PlasmaDerm®VU‐2010): Results of a Monocentric, Two‐Armed, Open, Prospective, Randomized and Controlled Trial (NCT01415622),” Journal of the European Academy of Dermatology and Venereology 29, no. 1 (2015): 148–155. 24666170 10.1111/jdv.12490

[60] Y. Zheng , Z. Zhuang , R. Zhou , et al., “Next‐Generation Oral Ulcer Management: Integrating Cold Atmospheric Plasma (CAP) With Nanogel‐Based Pharmaceuticals for Inflammation Regulation,” Advanced Healthcare Materials 14, no. 6 (2025): 2403223. 10.1002/adhm.202403223 39901375

[61] R. M. Garcia , B. T. Hardy , M. J. Kraay , and V. M. Goldberg , “Revision Total Knee Arthroplasty for Aseptic and Septic Causes in Patients With Rheumatoid Arthritis,” Clinical Orthopaedics & Related Research 468, no. 1 (2010): 82–89. 19727993 10.1007/s11999-009-1061-x PMC2795816

[62] S. M. Kurtz , K. L. Ong , J. Schmier , et al., “Future Clinical and Economic Impact of Revision Total Hip and Knee Arthroplasty,” supplement, Journal of Bone and Joint Surgery‐American 89, no. Suppl 3 (2007): 144–151. 10.2106/JBJS.G.00587 17908880

[63] R. Sarvilinna , H. S. A. Huhtala , T. J. S. Puolakka , J. K. Nevalainen , and K. J. J. Pajama ki , “Periprosthetic Fractures in Total Hip Arthroplasty: An Epidemiologic Study,” International Orthopaedics 27, no. 6 (2003): 359–361. 12898199 10.1007/s00264-003-0493-2 PMC3461876

[64] D. Stanley , I. Stockley , and C. Getty , “Simultaneous or Staged Bilateral Total Knee Replacements in Rheumatoid Arthritis. A Prospective Study,” Journal of Bone and Joint Surgery. British Volume 72, no. 5 (1990): 772–774. 2211753 10.1302/0301-620X.72B5.2211753

[65] H. Lindahl , H. Malchau , A. Odén , and G. Garellick , “Risk Factors for Failure After Treatment of a Periprosthetic Fracture of the Femur,” Journal of Bone and Joint Surgery. British Volume 88B, no. 1 (2006): 26–30. 10.1302/0301-620X.88B1.17029 16365115

[66] M. Yan , P. Hartjen , M. Gosau , et al., “Effects of a Novel Cold Atmospheric Plasma Treatment of Titanium on the Proliferation and Adhesion Behavior of Fibroblasts,” International Journal of Molecular Sciences 23, no. 1 (2022): 420. 10.3390/ijms23010420 PMC8745755 35008846

[67] L. M. Antonini , A. S. Takimi , V. P. Amaral , M. Camassola , and C. de Fraga Malfatti , “Cold‐Plasma‐Sterilized Nanostructured Ti6Al4V: Effect on Nanostructured Surface Morphology and Osteogenic Differentiation of Bone‐Marrow‐Derived Mesenchymal Stem Cells,” Journal of Materials Engineering and Performance 30, no. 10 (2021): 7236–7246.

[68] Y. T. Xie , Q. Wang , Z. Lin , et al., “The Effects of Air Cold Atmospheric Plasma on Cellular Early Attachment, Proliferation and Migration on Pure Titanium Surfaces,” Science of Advanced Materials 11, no. 10 (2019): 1392–1401.

[69] F. İbiş and U. K. Ercan , “Inactivation of Biofilms in Endotracheal Tube by Cold Atmospheric Plasma Treatment for Control and Prevention of Ventilator‐Associated Pneumonia,” Plasma Processes and Polymers 17, no. 10 (2020): e2000065.

[70] M. Modic , J. Kovač , J. R. Nicholls , et al., “Targeted Plasma Functionalization of Titanium Inhibits Polymicrobial Biofilm Recolonization and Stimulates Cell Function,” Applied Surface Science 487 (2019): 1176–1188.

[71] A. Przekora , J. Pawlat , P. Terebun , et al., “The Effect of Low Temperature Atmospheric Nitrogen Plasma on MC3T3‐E1 Preosteoblast Proliferation and Differentiation In Vitro,” Journal of Physics D: Applied Physics 52, no. 27 (2019): 275401.

[72] Y. Yang , J. Guo , X. Zhou , et al., “A Novel Cold Atmospheric Pressure Air Plasma Jet for Peri‐Implantitis Treatment: An In Vitro Study,” Dental Materials Journal 37, no. 1 (2018): 157–166. 29176301 10.4012/dmj.2017-030

[73] H. Xu , Z. Wei , Y. Huang , et al., “Enhancement of the Drug Sensitization of Cancer Cells by Plasma‐Activated Saline,” Plasma Processes and Polymers 20, no. 9 (2023): e2300001.

[74] R. Mentheour and Z. Machala , “Coupled Antibacterial Effects of Plasma‐Activated Water and Pulsed Electric Field,” Frontiers in Physics 10 (2022): 895813.

[75] Y. Li , T. Tang , H. J. Lee , and K. Song , “Selective Anti‐Cancer Effects of Plasma‐Activated Medium and Its High Efficacy With Cisplatin on Hepatocellular Carcinoma With Cancer Stem Cell Characteristics,” International Journal of Molecular Sciences 22, no. 8 (2021): 3956. 33921230 10.3390/ijms22083956 PMC8069277

[76] M. Adhikari , N. Kaushik , B. Ghimire , et al., “Cold Atmospheric Plasma and Silymarin Nanoemulsion Synergistically Inhibits Human Melanoma Tumorigenesis via Targeting HGF/c‐Met Downstream Pathway,” Cell Communication and Signaling 17 (2019): 52. 31126298 10.1186/s12964-019-0360-4 PMC6534917

[77] B. Haertel , F. Volkmann , T. von Woedtke , and U. Lindequist , “Differential Sensitivity of Lymphocyte Subpopulations to Non‐Thermal Atmospheric‐Pressure Plasma,” Immunobiology 217, no. 6 (2012): 628–633. 22130036 10.1016/j.imbio.2011.10.017

[78] G. Chen , Z. Chen , Z. Wang , et al., “Portable Air‐Fed Cold Atmospheric Plasma Device for Postsurgical Cancer Treatment,” Science Advances 7, no. 36 (2021): eabg5686. 34516919 10.1126/sciadv.abg5686 PMC8442862

[79] C. Almeida‐Ferreira , R. Silva‐Teixeira , M. Laranjo , et al., “Open‐Air Cold Plasma Device Leads to Selective Tumor Cell Cytotoxicity,” Applied Sciences 11, no. 9 (2021): 4171.

[80] N. K. Kaushik , N. Kaushik , P. Bhartiya , L. N. Nguyen , and E. H. Choi , “Glycolytic Inhibitor Induces Metabolic Crisis in Solid Cancer Cells to Enhance Cold Plasma‐Induced Cell Death,” Plasma Processes and Polymers 18, no. 5 (2021): 2000187.

[81] D. Sersenová , et al., “The Effect of Plasma Activated Medium and PBS on Human Melanoma Cells Compared With Other Cancer and Normal Cells,” Preprints 1 (2021): 2021010068.

[82] P. S. G. Subramanian , A. Jain , A. M. Shivapuji , N. R. Sundaresan , S. Dasappa , and L. Rao , “Plasma‐Activated Water From a Dielectric Barrier Discharge Plasma Source for the Selective Treatment of Cancer Cells,” Plasma Processes and Polymers 17, no. 8 (2020): e1900260.

[83] C. Li , H. Cheng , Z. Zhuang , et al., “FlexiPlasma Microcatheter–Embolic Material (FPM‐EM) Platform: A Non‐Inflammatory Pyroptosis Strategy for Precision Hepatocellular Carcinoma Therapy,” Small Methods n/a, no. n/a (2025): 2500231. 10.1002/smtd.202500231 40285389

[84] A. Chuangsuwanich , T. Assadamongkol , and D. Boonyawan , “The Healing Effect of Low‐Temperature Atmospheric‐Pressure Plasma in Pressure Ulcer: A Randomized Controlled Trial,” International journal of lower extremity wounds 15, no. 4 (2016): 313–319. 27581113 10.1177/1534734616665046

[85] B. Stratmann , T. C. Costea , C. Nolte , et al., “Effect of Cold Atmospheric Plasma Therapy vs Standard Therapy Placebo on Wound Healing in Patients With Diabetic Foot Ulcers: A Randomized Clinical Trial,” JAMA Network Open 3, no. 7 (2020): e2010411. 32672829 10.1001/jamanetworkopen.2020.10411 PMC7366186

[86] J. Gao , L. Wang , C. Xia , et al., “Cold Atmospheric Plasma Promotes Different Types of Superficial Skin Erosion Wounds Healing,” International wound journal 16, no. 5 (2019): 1103–1111. 31207094 10.1111/iwj.13161 PMC7949367

[87] M. Moelleken , F. Jockenhöfer , C. Wiegand , J. Buer , S. Benson , and J. Dissemond , “Pilot Study on the Influence of Cold Atmospheric Plasma on Bacterial Contamination and Healing Tendency of Chronic Wounds,” JDDG: Journal der Deutschen Dermatologischen Gesellschaft 18, no. 10 (2020): 1094–1101. 10.1111/ddg.14294 32989866

